# Performance-Based Measurement of eHealth Literacy: Systematic Scoping Review

**DOI:** 10.2196/44602

**Published:** 2023-06-02

**Authors:** Bradley Crocker, Olivia Feng, Lindsay R Duncan

**Affiliations:** 1 Department of Kinesiology & Physical Education McGill University Montreal, QC Canada

**Keywords:** eHealth literacy, measurement, performance-based, eHealth Literacy Scale, eHEALS, scoping review, review method, health literacy, library science, librarian, search strategy

## Abstract

**Background:**

eHealth literacy describes the ability to locate, comprehend, evaluate, and apply web-based health information to a health problem. In studies of eHealth literacy, researchers have primarily assessed participants’ perceived eHealth literacy using a short self-report instrument, for which ample research has shown little to no association with actual performed eHealth-related skills. Performance-based measures of eHealth literacy may be more effective at assessing actual eHealth skills, yet such measures seem to be scarcer in the literature.

**Objective:**

The primary purpose of this study was to identify tools that currently exist to measure eHealth literacy based on objective performance. A secondary purpose of this study was to characterize the prevalence of performance-based measurement of eHealth literacy in the literature compared with subjective measurement.

**Methods:**

We conducted a systematic scoping review of the literature, aligning with the PRISMA-ScR (Preferred Reporting Items for Systematic Reviews and Meta-Analyses extension for Scoping Reviews) checklist, in 3 stages: conducting the search, screening articles, and extracting data into a summary table. The summary table includes terminology for eHealth literacy, description of participants, instrument design, health topics used, and a brief note on the evidence of validity for each performance-based measurement tool. A total of 1444 unique articles retrieved from 6 relevant databases (MEDLINE; PsycINFO; CINAHL; Library and Information Science Abstracts [LISA]; Library, Information Science & Technology Abstracts [LISTA]; and Education Resources Information Center [ERIC]) were considered for inclusion, of which 313 (21.68%) included a measure of eHealth literacy.

**Results:**

Among the 313 articles that included a measure of eHealth literacy, we identified 33 (10.5%) that reported on 29 unique performance-based eHealth literacy measurement tools. The types of tools ranged from having participants answer health-related questions using the internet, having participants engage in simulated internet tasks, and having participants evaluate website quality to quizzing participants on their knowledge of health and the web-based health information–seeking process. In addition, among the 313 articles, we identified 280 (89.5%) that measured eHealth literacy using only a self-rating tool.

**Conclusions:**

This study is the first research synthesis looking specifically at performance-based measures of eHealth literacy and may direct researchers toward existing performance-based measurement tools to be applied in future projects. We discuss some of the key benefits and drawbacks of different approaches to performance-based measurement of eHealth literacy. Researchers with an interest in gauging participants’ actual eHealth literacy (as opposed to perceived eHealth literacy) should make efforts to incorporate tools such as those identified in this systematic scoping review.

## Introduction

### Background

Individuals form beliefs, make decisions, and enact behavior based on what they perceive to be high-quality and relevant information [1]. Ideas and beliefs about healthy and unhealthy behavior are shaped from various sources, but, above all, the internet plays an increasingly prominent role in people’s information diet regarding health-related topics. Research indicates that the internet is the first source people turn to for health information [2] and in some cases is deemed more credible than physician diagnoses [3]. The internet provides several affordances for accessing health information that cannot be matched by other resources: it is available 24 hours a day, queries are answered immediately, queries can be made anonymously, multiple points of view can be accessed and considered by the information seeker, and all of this is offered in the comfort of one’s own home [4]. Improved public access to health-related information concerning treatment and prevention has the potential to make a dramatic positive impact on public health outcomes. However, the realization of this positive impact on health-related behavior largely depends on how the internet is used by the information seeker.

Although the affordances of the internet as a health information resource are plentiful, locating high-quality health information on the internet can be challenging. The volume of health information on the web is massive and perpetually increasing; for example, a Google search for “sore throat treatment” returns nearly 2 billion results. Within this plethora of information, users’ search results are often clouded with misleading, inaccurate, or unsubstantiated information [5]; for example, in a sample of 227 web pages related to “immunity boosting” and “coronavirus,” Rachul et al [6] found that 85.5% of the sources portrayed “immune boosting” as beneficial for preventing COVID-19 infection despite no existent scientific evidence. Information seekers are tasked with sifting through search engine results to locate something relevant, trustworthy, and in an accessible format to inform their decisions. Norman and Skinner [7] describe this ability as eHealth literacy, which they define as “the ability to seek, find, understand, and appraise health information from electronic sources and apply the knowledge gained to addressing or solving a health problem” [7].

The construct of eHealth literacy was initially derived from 6 underlying foundational literacies: traditional literacy, information literacy, media literacy, health literacy, computer literacy, and scientific literacy [7]. Norgaard et al [8] extended this model further to identify 7 domains that ultimately contribute to an individual’s eHealth literacy: ability to process information (locating, interpreting, and applying health information), engagement in own health (interest in, and basic knowledge of, personal health and health care systems), ability to actively engage with digital services (competency with technology and navigating the internet), feel safe and in control (confidence and knowledge of securing personal health information), motivated to engage with digital services (accepting attitude toward web-based health resources and services), access to digital services that work (having the hardware and software to access web-based health resources and services), and digital services that suit individual needs (web-based health resources exist that are accessible and understandable to the individual user). Taken together, these models of eHealth literacy depict the diverse combination of critical skills needed to effectively acquire health information on the web.

Concurrent with their publication coining eHealth literacy, Norman and Skinner [9] published the eHealth Literacy Scale (eHEALS), an 8-item questionnaire wherein each item is rated using a 5-point Likert scale ranging from 1 (strongly disagree) to 5 (strongly agree), with higher scores indicating higher perceived eHealth literacy. In a systematic review of eHealth literacy measurement tools, Karnoe and Kayser [10] noted that this was the only tool used in multiple studies at the time of publication, and it remains the most widely used instrument across the literature to assess eHealth literacy [11]. As a short self-report assessment tool, the eHEALS is attractive for health care providers looking to optimize clinical efficiency and convenient for researchers to minimize participant burden. However, the self-report nature of this measure may limit its usefulness at providing an accurate depiction of how well people critically engage with web-based health information in today’s internet landscape.

Although self-report measurements have functioned as invaluable research tools in several settings, the efficacy of using self-report to evaluate one’s own skills, abilities, or proficiency in many contexts has been criticized for decades [12,13]. In their seminal review and meta-analysis of self-evaluations of ability across 55 studies, Mabe and West [12] found only a very modest (mean *r*=0.29) relationship between self-reported ability and performance-based measurement. Several psychological mechanisms may contribute to the considerable gap in estimates of ability and actual ability, many of which have been supported by research across a variety of cognitive skills [14]; for example, individuals have a tendency to be confident in skills where they feel fluent [14], meaning people may mistake the ability to fluidly perform a skill with the ability to perform that skill well. In the context of web-based health information seeking, people may mistake the ability to seamlessly locate some health information on the internet with the distinct ability to retrieve accurate, trustworthy, and relevant web-based health information. Many avid followers of the antivaccine movement have spent several hours surfing the internet for health-related information [15,16]. Their relatively high experience of web-based health information seeking may lead them to feel fluent in the skill, yet they are evidently not equipped to judge the quality of web-based information by the standards of evidence-based medicine.

Relating to web-based information seeking, ample literature indicates that people have a tendency to overestimate their ability with computers [17,18] as well as their ability to locate and understand information on the web [19,20]. Frequent overestimation of ability in this context may result from overlap between the cognitive skills necessary for the task itself and the metacognitive skills necessary for accurate self-assessment, meaning that those without the competence to consistently identify trustworthy information on the web are unlikely to have the capacity to recognize their lack of competence. Dunning [14] has put it more plainly: “If they had the skill to know they were making consistent mistakes, they would also have the skill to avoid those blunders in the first place.” Fittingly, in the context of web-based health information seeking, Stellefson et al [21] found that the least proficient searchers commonly exhibited high confidence in their searching abilities, indicating that self-efficacy is not a consistent predictor of eHealth literacy skills. Similarly, Maitz et al [22] found that adolescents who reported higher eHEALS scores did not perform better at selecting higher-quality websites to access health information. Illustrating this point further, Brown and Dickson [23] found that a group of occupational therapy graduate students reported lower average eHEALS scores than the high school participants in the original paper by Norman and Skinner [9]. Taken together, this literature indicates that intellectual hubris may play a bigger role in self-rated eHealth literacy than actual knowledge or ability; therefore, it should come as little surprise that a bulk of studies have noted no or little association between eHEALS scores and demonstrated eHealth skills [24-26]. Measures of eHealth literacy that involve more objective performance-based measurement of eHealth skills and abilities may be more promising at assessing how people actually behave on the web; however, such measures seem to be more scarcely used among researchers to date [11].

Although research syntheses have been undertaken related to eHealth literacy measurement instruments and their properties [10,27], no synthesis to date has looked specifically at performance-based measures of eHealth literacy. This work is needed to assist researchers in identifying performance-based measurement tools in the literature that may be applied in future projects.

### Objectives

The purposes of this study were 2-fold. The first purpose was to identify tools that currently exist to measure eHealth literacy based on objective performance. Within this purpose, we were interested in the design of these tools, their intended population, and whether their authors included some evidence of validity. The second purpose of this study was to characterize the prevalence of performance-based eHealth literacy measurement tools in the literature broadly compared with subjective eHealth literacy measurement tools.

## Methods

### Overview

We conducted a systematic scoping review of the literature. A scoping review was deemed appropriate for the purpose of this study, given its broad focus on how research involving eHealth literacy measurement is conducted [28]. This scoping review was conducted in 3 main stages, aligning with the PRISMA-ScR (Preferred Reporting Items for Systematic Reviews and Meta-Analyses extension for Scoping Reviews) checklist [29].

### First Stage

In the first stage, we devised an electronic search protocol that was completed by 2 researchers. We used the following search string, modified from the one used in a systematic review conducted by Karnoe and Kayser [10]:

(“eHealth literacy” OR “electronic health literacy” OR “e-health literacy” OR “digital health literacy” OR (“health literacy” AND “digital literacy”) OR (“health literacy” AND “computer literacy”)) AND (scale OR measure OR survey OR questionnaire OR test OR assessment)

We applied this string to search 6 relevant databases (MEDLINE; PsycINFO; CINAHL; Library and Information Science Abstracts [LISA]; Library, Information Science & Technology Abstracts [LISTA]; and Education Resources Information Center [ERIC]) selected in consultation with a university librarian. All searches were conducted in June 2021. Across these 6 databases, 1735 search results were obtained, which we reduced to 1444 (83.23%) unique articles after removing duplicates.

### Second Stage

In the second stage, we conducted 3 screening phases to narrow down the search results. In each screening phase, articles were sought that met the following inclusion criteria: (1) written in English; (2) published in peer-reviewed journals; and (3) created, revised, or used an eHealth literacy assessment tool (in accordance with the definition of eHealth literacy proposed by Norman and Skinner [7], quoted earlier in this paper). The first screening phase involved screening all articles using their titles, which was carried out by the first author. After this phase, of the 1444 articles, 788 (54.57%) were retained for possible inclusion. The second screening phase involved screening all remaining articles using their abstracts, which was carried out by the first and second authors. If either author opted to include the article, it was kept for the third screening phase. After this phase, of the 788 articles, 374 (47.5%) were retained for possible inclusion. The third screening phase involved reading the full text of all remaining articles, which was carried out by the first and second authors. During this phase, each author also classified each included article as containing only a subjective eHealth literacy measure (based on self-rated assessment) or as containing a performance-based eHealth literacy measure (based on practical skill or knowledge assessment).

**Figure 1:**
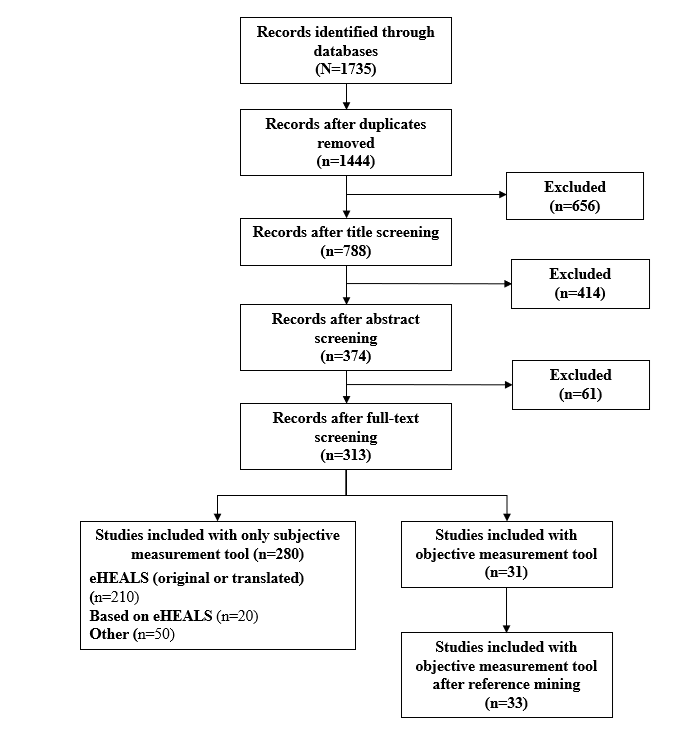
Diagram summary of the systematic scoping review process.

After the third screening phase, of the 374 articles, 311 (83.2%) were retained for inclusion in this scoping review; 31 (10%) of these 311 articles contained a performance-based measure of eHealth literacy. In cases of disagreement between the first and second authors during the third screening phase, the third author was consulted, and discussion ensued until consensus was reached. Throughout the entire screening process, all authors only excluded articles that very evidently did not meet the inclusion criteria. At this point, the first author read the bibliographies of the articles containing a performance-based measure of eHealth literacy (n=31) and highlighted titles that could meet the inclusion criteria of this scoping review. After discarding studies that had already been considered in the screening process, new studies were selected (n=6) and read in full by the first and second authors. Of these 6 new studies, 2 (33%) were included in the scoping review, and both contained a performance-based measure of eHealth literacy. Thus, the total number of articles included in this scoping review is 313, of which 33 (10.5%) contain a performance-based measure of eHealth literacy. The scoping review process we undertook is summarized in Figure 1.

### Third Stage

In the third stage, we extracted data from each of the articles containing a performance-based measure and summarized them into a table. The first and second authors each reread the articles containing a performance-based measure of eHealth literacy (n=33) and input information from each into a comprehensive table devised by all 3 authors. This table was later simplified into appropriate headings, selected in the interest of providing a concise and relevant summary of the findings in this paper. In judging evidence of validity, we considered 3 relevant types of validity: ecological validity, criterion validity, and construct validity. Ecological validity describes the extent to which a measure represents or predicts behavior in real-world settings [30]. In the context of measuring performance-based eHealth literacy, we considered a measure to be ecologically valid if it involved participants accessing real or authentically simulated websites to gather or evaluate health information. Criterion validity describes the extent to which the scores of a measure predict scores of another established measure of interest [30]. We stated that a measure had criterion validity if the authors found statistically significant correlations between scores of their objective eHealth literacy measure and scores from another relevant construct or validated measure (eg, higher scores on this measure correlate with higher scores of another validated measure of computer-related skills). Construct validity describes the extent to which the scores of a measure reflect the actual phenomena that measure intends to assess [30]. This concept has some overlap with criterion validity; however, for our purposes, we stated that a measure had construct validity if meaningful differences were reported based on relevant lifestyle or demographic factors or reported among groups that can be reasonably assumed to have different levels of eHealth literacy (eg, those with more health-related education score consistently higher). We also noted whether the performance-based measurement tool described in the article was included in its full form, partial form (examples provided but some parts omitted), or not at all.

## Results

### Performance-Based eHealth Literacy Measurement Tools

Our scoping review identified 33 peer-reviewed studies using a tool to measure eHealth literacy that incorporated a performance-based aspect. Within these studies, there were just 2 measures used more than once. In total, 3 (9%) of the 33 articles used the eHealth literacy assessment toolkit (eHLA) created by Karnoe et al [31], and another 3 (9%) used the Research Readiness Self-Assessment created by Ivanitskaya et al [32]. We thus identified a total of 29 unique performance-based measures of eHealth literacy. It is notable that many of these studies use differing terminology to describe the construct of eHealth literacy, as outlined in Table 1; however, all studies included in this review measure constructs aligning with the definition of eHealth literacy originally proposed by Norman and Skinner [7] based on our judgment.

We categorized the 29 unique performance-based measurement tools identified in this scoping review into 5 broad categories according to the structure of their main measurement technique. It should be noted that several of the measurement tools have components that fall into ≥2 of these categories; for instance, the Research Readiness Self-Assessment-health (RRSA-h) [53,55] measures declarative knowledge about the internet and has participants respond to questions that simulate using web-based health information. We have described the instrument design of each performance-based measurement tool in Table 1 such that readers can gain a fuller understanding of each tool compared with the brief descriptions within the body of this paper.

**Table 1:** Summary of included articles containing a performance-based measure of eHealth literacy (organized alphabetically by category).

Authors, year		Terminology for measured construct	Participants and context	Instrument design	Health topics	Evidence of validity	Instrument availability
**Answering health** **-related questions using the internet**							
	Agree et al [33], 2015	Web-based health literacy	323 participants aged 35 to 90 years	Six health-related questions were answered by performing web-based searches on a computer, limited to 15 minutes per task; answers were coded by 2 researchers for response accuracy (0 or 1) and specificity (0, 1, or 2) to give a score ranging from 0 to 18	Diet and nutrition guidelines, skin cancer, alternative medicine, vaccines, assistive health technology, and over-the-counter genetic testing	Construct validity was demonstrated in that having a college degree and daily internet use were positively associated with more successful health information searches, and the oldest age group had lower success scores compared with younger participants; criterion validity was demonstrated in that higher health literacy was positively associated with success on some search tasks	Partially
	Blakemore et al [34], 2020	eHealth literacy	A massive open web-based course run 8 times	Participants responded to 1 health-related question and were asked to list the resources they used to inform that answer; answers were coded according to the extent that quality resources were used in this question	Epigenetics and cancer	Ecological validity was demonstrated via having participants access web-based resources to inform answers to a health-related question	Yes
	Chang et al [35], 2021	Searching performance	11 older adult participants	Participants were asked to search for specific health information using a web browser on a computer; search completion time and problem correctness were measured by researchers during live observation	Vaccination for older adults, stroke, and angina	Ecological validity was demonstrated via participants accessing real-world web-based health information to answer health-related questions	No
	Freund et al [36], 2017	eHealth literacy	79 older adult participants	Participants were asked to answer 6 questions (3 each for 2 health scenarios) while being given the option of using links to web-based medical databases with relevant information	Hypertension, high blood pressure, osteoporosis, breast cancer, and prostate cancer	Construct validity was demonstrated in that test scores for the intervention group improved; ecological validity was demonstrated in that participants responded to questions by referencing a web-based resource	Yes
	Kordovski et al [37], 2020	eHealth search skills	56 undergraduate students enrolled in psychology courses	Participants were instructed to find the answer to 5 short questions and 1 vignette-based question using an internet browser of their choice; participants’ accuracy, time to complete each task, and total number of search queries were recorded	Headaches, migraines, and Lyme disease	Criterion validity was demonstrated in that long-answer accuracy was associated with better performance on a learning and memory composite test; construct validity was demonstrated in that lower performance on short questions was associated with lower maternal education and lower socioeconomic status	Yes
	Loda et al [38], 2020	Information-seeking behavior	140 medical students	Students were randomly assigned to use a specific search engine (Google, Medisuch, or free choice) and had 10 minutes to fill in a worksheet outlining a diagnostic recommendation; to pass, students needed to give at least 1 of 3 recommendations matching those of a clinical expert	Histamine intolerance	None	No
	Quinn et al [25], 2017	eHealth literacy	54 adults	Participants were presented 6 health questions, and they used a browser to search for information to answer the questions; each answer was scored as correct or incorrect, with a final sum score out of 6	Various topics	Criterion validity was demonstrated because the scores correlated with health literacy	Yes
	Sharit et al [39], 2008	Internet search task performance	40 older adults	Participants were assigned 6 search problems involving health-related information, for which they had to provide an answer using information they found on the internet; participants had 15 minutes to solve each problem, and the problems were progressively more complex; the problem solutions were scored as incorrect, partially correct, or correct by the researcher to create a task performance score; scores were weighted by difficulty, and participants’ completion times for each problem were also measured and factored into the score such that faster times indicated better performance	Various	Criterion validity was demonstrated in that higher performance correlated with higher knowledge of the internet, as well as with measures of reasoning, working memory, and perceptual speed	Yes
	Sharit et al [40], 2015	Search accuracy	60 adults	Participants were given a health scenario, followed by a series of questions related to it, and they could use the internet to find answers; to assess accuracy, researchers assigned a score for each question; questions were weighted based on their difficulty (differed in complexity and number of subtasks)	Multiple sclerosis	Criterion validity was demonstrated as search accuracy significantly correlated with reasoning, verbal ability, visuospatial ability, processing speed, and executive function	Yes
	van Deursen and van Dijk [41], 2011	Internet skills performance	88 adults	Participants completed 9 health-related assignments using a computer with high-speed internet; assignment was deemed successfully completed if a correct answer was provided and deemed unsuccessful if no correct answer was provided in the given time frame	Various	Ecological validity was demonstrated via participants using unrestricted web-based searching to answer health-related questions	Yes
	van Deursen [42], 2012	Internet skills performance	88 adults	Participants completed 9 health-related assignments using a computer with high-speed internet; assignment was deemed successfully completed if a correct answer was provided and deemed unsuccessful if no correct answer was provided in the given time frame	Various	Ecological validity was demonstrated via participants using unrestricted web-based searching to answer health-related questions; construct validity was demonstrated as education was predictive for making incorrect decisions based on the information found	Yes
**Simulated internet tasks**							
	Camiling [43], 2019	Actual eHealth literacy (distinct from perceived eHealth literacy)	40 grade-10 students from public and private schools	Participants completed 10 simulation tasks; 2 researchers used a rubric to rate eHealth literacy based on task performance	Not specified	Ecological validity was demonstrated via use of simulated internet research tasks resembling a realistic environment	No
	Chan and Kaufman [44], 2011	eHealth literacy	20 adult participants aged between 18 and 65 years	Participants completed eHealth tasks while verbalizing their thoughts (think-aloud protocol); researchers observed their performance, rated accuracy, and denoted barriers based on video capture, audio recording, and notes taken during observation	Comparing hospital ratings	Ecological validity was demonstrated via participants actively completing health-related internet tasks in a realistic environment	Partially
	Maitz et al [22], 2020	Health literacy (the authors note in their study that their understanding of health literacy includes “internet-based information literacy and reading literacy”)	14 secondary school students aged 12 to 14 years	Participants were asked to provide health-related advice in response to a short narrative text; they were asked to take screenshots of all searches and web pages opened; the web pages were later classified by researchers as good, fair, poor, or bad	Rhinoplasty and skin cancer	None	Yes
	Neter and Brainin [24], 2017	eHealth literacy	88 older adults	Participants completed 15 computerized simulation tasks assessing digital and health literacy skills; tasks were rated as completed or not completed by the researcher upon reviewing the recorded performance; time needed to perform the task was also recorded; 2 researchers provided overall observational judgment on participants’ performance, ranging from 1 (poor) to 5 (good); a third researcher evaluated whether disagreements were present	Various topics	Construct validity was demonstrated because lower performers had significantly fewer years of experience using the internet	Yes
	van der Vaart et al [26], 2011	eHealth literacy	88 adults	Participants completed 9 health-related assignments using a computer with high-speed internet; assignment was deemed successfully completed if a correct answer was provided and deemed unsuccessful if no correct answer was provided in the given time frame	Various	Ecological validity was demonstrated via participants using unrestricted web-based searching to answer health-related questions	Yes
	van der Vaart et al [45], 2013	eHealth literacy	31 adult patients	In study 1, participants could use the internet freely to complete 6 health-related assignments; in study 2, participants used specific websites to complete 5 health-related assignments; researchers coded whether the assignment was completed and whether help was needed; in addition, the time needed to perform each assignment was recorded; the performance was ultimately scored as good, reasonable, or poor according to the skills participants used to execute the assignment	Various	Construct validity was demonstrated through correlations of higher performance with higher education	Yes
	Witry et al [46], 2018	eHealth task performance	100 adult patients with COPD^a^	Participants completed a series of timed eHealth simulation exercises using a laptop computer and 2 different tablet devices; the time taken to complete each task was measured and used to indicate task performance, with faster times indicating better performance	COPD	Construct validity was demonstrated because those who reported using video chat took less time than nonusers to complete most of the tasks	No
**Website evaluation tasks**							
	Kalichman et al [47], 2006	Health information evaluation skills	448 adults who used the internet <3 times in the month before screening	Participants rated 2 preselected web pages—1 from a medical association and 1 with scientifically unsupported claims—on 5 dimensions of website quality; a larger difference in scores indicated higher health information evaluation skills	HIV and AIDS treatment	Construct validity was demonstrated in that those receiving internet skills training had better discrimination	Yes
	Mitsuhashi [48], 2018	eHealth literacy evaluation skills	300 adult participants	Participants were shown a search engine results page with 5 websites and asked which should be viewed first; the list included 2 commercial websites, 2 personal health care websites, and 1 government website; participants choosing the government website were assigned 1 point, others were assigned 0 points	Not specified	Construct validity was demonstrated in that evaluation skills improved significantly in an e-learning intervention group compared with the control group	No
	Schulz et al [49], 2021	Health literacy	362 adults	Participants rated 2 health information websites (one was of high quality, whereas the other was of low quality) using 3 seven-step semantic differential scales; in addition, participants were asked to choose beneficial depression treatments from a list of relevant and nonrelevant treatments	Depression treatment	Criterion validity was demonstrated in that those with high health literacy and accurate recognition of the low-quality website demonstrated good judgment for depression treatment	Partially
	Trettin et al [50], 2008	Website evaluation	142 high school students	Two measures: first, a brief 2-item pretest of knowledge about how to evaluate a website, and second, participants ranked 2 different websites (assigned to them from a list of 12 websites) using 6 credibility factors, with scores ranging from 1 (very bad) to 5 (very good)	Not specified	Ecological validity was demonstrated in that participants ranked authentic health-related websites according to their credibility	Yes
	Xie [51], 2011	eHealth literacy	124 older adults	Participants were asked to evaluate the quality of 20 health websites: 10 selected from the Medical Library Association’s recommended sites and 10 from a commercial search engine; each correct assessment received 1 point, whereas incorrect or uncertain assessments received 0 points	Not specified	Construct validity was demonstrated because scores improved after an educational intervention	No
**Knowledge of the** **web-based** **health information–seeking process**							
	Hanik and Stellefson [52], 2011	eHealth literacy	77 undergraduate health education majors	Researchers used the RRSA-h^b^, which is a questionnaire that tests participants’ declarative knowledge of concepts, skills, and thinking strategies related to using the internet to find health information	Various	Ecological validity was demonstrated in the study by Ivanitskaya et al [53]	No
	Hanna et al [54], 2017	eHealth literacy	165 adult dental patients	Participants were asked to circle the web-based health information quality seals they recognized and report the purpose of 1 circled figure	Third molar knowledge	Criterion validity was demonstrated in that the eHEALS^c^ scores correlated with the dental procedural web-based information–seeking measure; construct validity was demonstrated in that web-based dental procedural information seeking was significantly associated with educational attainment and dental decisional control preference	Yes
	Ivanitskaya et al [53], 2006	Health information competency	400 college-aged students	Researchers used the RRSA-h, which is a web-based quiz that assesses declarative and procedural knowledge related to web-based health information seeking	Various	Ecological validity was demonstrated in that some questions had participants access real health-related websites to assess their credibility	No
	Ivanitskaya et al [55], 2010	eHealth literacy skills	1914 undergraduate and graduate students enrolled in health-related courses	Researchers used the RRSA-h, which is a web-based quiz that assesses declarative and procedural knowledge related to web-based health information seeking; a proxy measure of critical judgment skills related to pharmacies was also included	Pharmaceuticals and various others	Construct validity was demonstrated in that evaluation skills positively correlated with the number of earned college credits and being enrolled in a health-related major	Partially
	St. Jean et al [56], 2017	Digital health literacy	19 adolescents	Participants were given 13 questions related to searching for health information; researchers analyzed responses using thematic analysis; no evident scoring system used	Type 1 diabetes	None	Yes
	van der Vaart and Drossaert [57], 2017	Digital health literacy, eHealth literacy	200 adults	Participants completed a 28-item questionnaire: 21 are self-report items, whereas 7 are performance-based items for each of which there is a correct answer	Various	None for performance-based items	Yes
**Health-related knowledge**							
	Holt et al [58], 2019	eHealth literacy	246 adult patients with diabetes, other endocrine conditions, and gastrointestinal diseases	Researchers used eHLA^d^ performance tests (tools 1 and 4); tool 1 is a performance-based health literacy test based on an information leaflet, and tool 4 is a performance test for knowledge of health and health care	Various	Construct validity was demonstrated in that educational level was positively correlated with tool 4	Partially
	Holt et al [59], 2020	eHealth literacy	366 nursing students	Researchers used eHLA performance tests (tools 1 and 4); tool 1 is a performance-based health literacy test based on an information leaflet, and tool 4 is a performance test for knowledge of health and health care	Various	Construct validity was demonstrated in that graduate-level students scored higher than entry-level students, and performance on tools 1 and 4 was correlated with having at least 1 parent with experience in the social or health care system	Partially
	Karnoe et al [31], 2018	eHealth literacy	475 adults used as a validation sample	Researchers used the eHLA, which consists of 7 tools, 2 of which are objective measures: tool 1 is a performance-based health literacy test based on an information leaflet, and tool 4 is a performance test for knowledge of health and health care	Various	None	Partially
	Liu et al [60], 2020	Digital health literacy	1588 adult participants	Participants were provided 5 randomly selected items from a large web-based health information bank and asked whether the information was right or wrong; 2 items were designed to be relatively easy to judge accurately, 2 were moderately easy, and 1 was difficult; participants scored 1 for each accurate judgment and 0 for being incorrect or unsure	Various	Ecological validity was demonstrated in that the web-based health information bank was generated from real web-based sources; construct validity was demonstrated because participants at high risk for misjudging health information had lower education level, poorer health, and used the internet less	Partially

^a^COPD: chronic obstructive pulmonary disease.

^b^RRSA-h: Research Readiness Self-Assessment-health.

^c^eHEALS: eHealth Literacy Scale.

^d^eHLA: eHealth literacy assessment toolkit.

### Answering Health-Related Questions Using the Internet

One of the most common types of performance-based measurements identified in this scoping review involved participants answering health-related questions using the internet; these were questions for which responses could be scored according to correctness, completeness, and specificity. In the majority of the studies using this assessment method participants were allowed open access to the internet to inform their answers. An exception was the study by Freund et al [36] in which participants were provided expert-checked links to helpful resources that they could choose to use for answering health-related questions. In addition to the correctness of participants’ responses, some researchers factored completion time into participant scores such that faster completion time indicated greater proficiency [39,40]. Chang et al [35] similarly considered faster completion times indicative of better web-based search performance, provided the responses were correct. The health questions posed in these measurement tools ranged from encompassing several diverse topics to being focused on 1 topic in depth; for example, both Agree et al [33] and Quinn et al [25] asked participants about 6 diverse health topics, whereas Kordovski et al [37] and Loda et al [38] asked participants to perform 1 in-depth medical diagnosis relating to symptoms of Lyme disease and histamine intolerance, respectively.

An advantage of eHealth literacy measurements where participants use the internet to answer health-related questions is that participants can be assessed without the need for researcher observation and video recording, except in cases where completion time is additionally factored into the scores. With these measurement tools, participants’ responses to questions were graded by ≥1 researchers according to a predetermined rubric. The majority of measurement tools in this category coded responses as simply *correct* (1 point) or *incorrect* (0 points); however, Agree et al [33] also examined specificity (on a scale ranging from 0 to 2 points) and Kordovski et al [37] gave partial credit (1 out of 2 points) for coming close to the correct response by diagnosing something similar to Lyme disease. In contrast to considering response correctness, Blakemore et al [34] gauged participants’ eHealth literacy skills based on whether they included a “detailed list of resources,” had “written about using resources,” or had no reference to web-based health resources in their response to a health-related question.

### Simulated Internet Tasks

Another common type of performance-based eHealth literacy assessment identified in this scoping review involved web-based information–seeking simulations, where participants’ web-based behavior was observed (either in real time or via video recording) and assessed for proficiency by researchers. Proficiency was determined by assessing whether participants were able to complete a set of tasks; many researchers went further by also assessing the degree of efficiency and *correctness* that the participants exemplified throughout each simulated task; for example, in the study by van der Vaart et al [26], participants’ task performance was simply coded as *successful* or *unsuccessful*, depending on whether they accomplished the end result of the task (eg, adding a specified website as a bookmark or downloading a specific image), whereas in the study conducted by Camiling [43], a rubric was developed and used to rate participants’ proficiency while researchers observed them completing 10 health-related tasks on an internet-connected computer. Similarly, Neter and Brainin [24] viewed recordings of participants completing 15 simulation tasks on an internet-connected computer and rated each task on whether it was completed and additionally on the quality of task performance on a scale ranging from 1 (poor) to 5 (good). Video and audio recordings were commonly used to record participants’ actions and thoughts in simulation-based measurement tools, with the notable exception of Maitz et al [22] who had participants record screenshots of the websites they visited.

eHealth literacy assessments based on simulated internet tasks tended to be the most time-consuming for participants: the 15 simulation tasks in the study by Neter and Brainin [24] took approximately 90 minutes for each participant to complete, and the 5 or 6 simulation tasks in the study by van der Vaart et al [45] took an approximate median time of 30 minutes for each participant to complete. A notable exception is the study by Witry et al [46] in which the assigned eHealth simulation tasks were far simpler and could be completed in 1 minute; however, it should be noted that participants’ completion time was the only metric used to gauge participants’ eHealth literacy in this study. It also stands to reason that evaluating the proficiency of participants’ recorded web-based behavior is quite time-consuming for researchers compared with assessment methods that are scored based on correct or incorrect responses to questions. In the studies conducted by Chan and Kaufman [44] as well as van der Vaart et al [26], researchers factored in the correctness of responses in addition to researcher-observed performance to gauge participants’ eHealth literacy, combining task simulation with the *Health-related questions using the internet* methods discussed previously.

### Website Evaluation Tasks

A third prominent type of performance-based eHealth literacy assessment identified in this scoping review involved participants evaluating the quality of health-related websites. In the studies conducted by Kalichman et al [47], Schulz et al [49], and Trettin et al [50], participants rated 2 different websites on 5, 7, and 6 dimensions related to their credibility, respectively. In each of these studies, the authors presented participants with a website of high quality and another of low quality, and participants’ eHealth literacy was determined based on whether they rated the websites accordingly (with a larger difference between high- and low-quality website ratings indicating greater proficiency).

Deviating from the structure of these studies but still related to evaluating website quality, Mitsuhashi [48] had participants select which website they should view first from a search results page with 5 options (2 commercial websites, 2 personal health care websites, and 1 government website), in which participants who selected the government website were said to have proficient evaluation skills. Xie [51] collected 20 websites to present to participants—10 from a commercial search engine and 10 from a medical association’s recommended websites—and had participants assess each website as high or low quality. Correct assessments earned participants 1 point, and incorrect or uncertain assessments earned 0 points, for a maximum score of 20.

The rating of the quality of real health-related websites by participants significant ecological validity in terms of participants’ knowledge of how to recognize signifiers of credible health information on the web; however, in contrast to the simulated internet task measurement tools, these tools do not require participants to form queries or extract information to apply to health-related problems. Although evaluating the credibility of a web-based source is undoubtedly a pivotal component of eHealth literacy, it does not encompass all components of eHealth literacy as defined by Norman and Skinner [7].

### Knowledge of the Web-Based Health Information–Seeking Process

Distinct from measurement tools where participants demonstrate eHealth literacy skills through task completion or by correctly answering health-related questions using the internet, other tools tested participants on their knowledge of procedural internet skills and information-seeking strategies to gauge their eHealth literacy. The most used tool in this category is the RRSA-h [53,55]. The RRSA-h uses multiple choice and true or false questions to assess participants’ declarative knowledge of web-based health information seeking, as well as some participative problem-based exercises to assess elements of their procedural knowledge. Ivanitskaya et al [53,55] as well as Hanik and Stellefson [52] used this measure to assess eHealth literacy skills in undergraduate and graduate students. The RRSA-h takes approximately 30 minutes on average to complete.

In contrast to this measure that takes relatively long to complete, Hanna et al [54] used just 1 item to gauge participants’ ability to recognize high-quality web-based information by asking them to circle web-based health information quality seals that they recognized from a set of 19 images. In addition, participants were asked to explain the purpose of one of the images they circled. Researchers analyzed whether participants were able to identify ≥1 real quality seals, as well as whether they could identify that the image was used to signify credibility. van der Vaart and Drossaert [57] used 7 performance-based multiple-choice quiz items to gauge 7 dimensions of participants’ eHealth literacy skills, which were added to supplement a subjective measure of eHealth literacy. Finally, in this category, St. Jean et al [56] devised a written assignment where adolescents advised a fictional peer about using the internet to find information about type 1 diabetes through 13 open-ended questions. The researchers did not use an evident scoring system to rate the eHealth literacy skills of this population; rather, they applied thematic analysis to characterize the eHealth literacy skills of the participant group as a whole.

Quizzing participants on their knowledge related to eHealth literacy does not require recording or observing participants’ web-based activity, and nor does it require necessarily providing participants with internet access, which makes this method more accessible for some research settings in terms of resources and complexity than other tools mentioned previously. As participants are not actively carrying out web-based health information–seeking tasks with these measurement tools, the tools may not carry the same degree of ecological validity as those mentioned previously. However, basing eHealth literacy assessment on participants’ knowledge of proficient web-based information–seeking skills still avoids many of the pitfalls related to subjective self-report measures of eHealth literacy.

### Health-Related Knowledge

We identified 2 measurement tools that examined participants’ baseline knowledge of health and their ability to apply that knowledge as an indicator of eHealth literacy. The most prominent tool is the eHLA [31], which is composed of 7 distinct measurement tools. Tools 1 and 4 within this set are performance-based measures that assess functional health literacy and participant knowledge of health and disease, respectively. The performance-based aspects of this tool (as well as its subjective tools) were used by Holt et al [58,59] to measure eHealth literacy in medical outpatients and nursing students.

Liu et al [60] also created a performance-based eHealth literacy measurement tool that assesses participants’ health knowledge. In their study, the authors generated a 310-item bank of examples of web-based health information, which included items labeled “easy,” “moderate,” and “difficult.” Participants were randomly assigned 5 items (2 easy, 2 moderately easy, and 1 difficult) and asked to rate the information as correct, incorrect, or unsure. Participants were given 1 point for accurately identifying information as correct or incorrect and 0 points otherwise for a score out of 5 representing their eHealth literacy.

In these 2 eHealth literacy measurement tools, there are no components that directly assess computer literacy in a performance-based manner nor do they contain performance-based components related to actively seeking health information (eg, forming a query or selecting a source). We have included them in this scoping review because the authors themselves define these as measures of eHealth literacy (and it should be noted that the eHLA includes subjective measurements that touch upon computer-related components of eHealth literacy). However, judging their performance-based components solely by the definition of eHealth literacy proposed by Norman and Skinner [7], these measures may be more accurately characterized as partial measures of the construct.

### Prevalence of Performance-Based eHealth Literacy Measurement Tools

Of the 313 studies included in this scoping review, 33 (10.5%) used a performance-based measurement tool, and 280 (89.5%) used measures of eHealth literacy that only incorporated subjective or self-rating aspects. Furthermore, it is notable that 210 (67.1%) of the 313 studies reported using eHEALS in either its original form or translated into another language. Our findings indicate that current research on eHealth literacy has a strong tendency to rely on self-rated perceptions of eHealth literacy rather than assessing actual ability.

## Discussion

### Principal Findings

The primary purpose of this scoping review was to identify and describe tools that measure eHealth literacy based on objective performance (as opposed to subjective self-rating). We identified 29 such measurement tools, of which only 2 had been used in >1 peer-reviewed study as of the date of our search. These measurement tools were the RRSA-h [55], which is a web-based quiz measuring declarative and procedural knowledge related to web-based health information seeking, and the eHLA [31], which assesses eHealth literacy through 7 distinct tools, of which 2 are performance based. It is noteworthy that the 2 performance-based tools within the eHLA do not touch upon computer- or internet-specific skills or knowledge; as such, not all aspects of eHealth literacy are measured in a performance-based fashion. The same critique may be applied to the measurement tool created by Liu et al [60] who similarly do not directly address computer literacy or media literacy in their performance-based assessment tool.

The second purpose of this scoping review was to characterize the prevalence of performance-based eHealth literacy measurement tools in the literature in contrast to subjective measurement tools. Our findings indicate that the vast majority of current research on eHealth literacy relies on self-rated perceptions of eHealth literacy rather than assessing actual ability. This is concerning, considering the limited utility of this short self-report measure for predicting performed eHealth literacy as indicated by a substantial body of literature [21,22,24-26]. If researchers are interested in gauging participants’ true ability to locate, evaluate, and use web-based health information, more efforts should be made to incorporate performance-based measurement tools of eHealth literacy, such as those identified in this scoping review.

Another notable finding of this scoping review is that of the 29 unique eHealth literacy measurement tools with performance-based components identified, only 2 had been used in >1 peer-reviewed study at the time of this research. This comes in stark contrast to the prevalence of eHEALS, which was used by 210 (67.1%) of the 313 studies in various contexts and languages. This could be due in part to several of the studies (8/33, 24%) not including full versions of their performance-based instruments, making it challenging for other researchers to replicate these measures in other projects. Another implicit challenge to creating a performance-based eHealth literacy measure that may be adopted for widespread use is the changing state of scientific consensus on health-related topics, meaning that *correct* answers to health-related questions may need to be updated over time; for example, dietary guidelines have changed substantially in the past few decades because they have been updated based on our growing scientific knowledge [61,62]. In addition, health-related topics can differ substantially in relevancy or saliency among different populations, meaning a performance-based eHealth literacy measure effective for a particular population may not be as useful for another; for example, Loda et al [38] designed a performance-based measure for use by medical students where they needed to produce a histamine-intolerance diagnosis on par with a clinical expert using the internet for research, which is a task that is likely beyond the abilities of typical users without comparable existing medical knowledge.

Judging these performance-based measurement tools by the definition of eHealth literacy proposed by Norman and Skinner [7], the tools with the greatest perceived ecological validity include those having participants answer health-related questions using the internet and those having participants engage in simulated web-based health–related tasks. These measurement tools evaluate participants’ web-based health information–seeking abilities in settings similar to the ones they encounter when seeking information on their own computers. The time-consuming nature (for both participants and researchers) and the equipment needs of these measures present substantial barriers to their use. Efforts to streamline some of these measurement tools by including only strictly necessary components could assist with broader use. One example of a concise measure is offered by Witry et al [46], who created a set of simple eHealth simulation tasks that could be completed in <1 minute; however, it should be noted that this measure assesses one’s ability to navigate an eHealth platform more so than to actively seek health information on the web. While considering the challenges to using performance-based eHealth literacy measures, researchers should also weigh the major advantages of these tools in producing a more accurate depiction of performed eHealth literacy compared with short self-report measures.

One glaring absence across most performance-based measures of eHealth literacy is any mention of social media. In discussing the modern utility of eHEALS, Norman [63] noted that the shifting nature of the internet, with everyday users routinely contributing information on the web as opposed to only accessing it, necessitates modifications to existing measurement tools to maintain validity. Consumers are more frequently turning to social media platforms with health-related queries, where they stand to gain social and emotional support from peers while gaining crowdsourced wisdom related to their health problem [64]. The validity and trustworthiness of information quality on these platforms has been flagged as a major concern [65]; however, public health organizations have also used these engaging platforms to distribute high-quality and up-to-date information [66,67]. As an acknowledgment that users are able to gain useful and accessible health information using social media, more eHealth literacy measurement tools should incorporate these platforms into their assessment. One example of an assessment tool with a social web-based component is provided in the study by van der Vaart et al [45], where participants were asked to demonstrate interacting with a health care rating website and peer support forum, for which their proficiency was assessed based on independent task completion and overall task performance. Given the prominence of social media in today’s web-based informational landscape, future performance-based measures of eHealth literacy should consider assessing participants’ ability to identify credible health information on social media platforms.

### Limitations

This study includes a few notable limitations. This scoping review only considered peer-reviewed journal articles published in English, which may have caused bias in our findings. Furthermore, it is possible that we missed including studies that evaluated skills under the umbrella of eHealth literacy but did not describe this using any of the terminology in our search protocol. We did locate studies using terms such as “health information evaluation skills” [47] and “information-seeking behavior” [38] through our search protocol, indicating some ability to detect studies deviating from our search terms. Finally, it should be noted that the evidence of validity provided in Table 1 provides only a limited surface-level judgment of 3 types of validity (criterion, construct, and ecological validity) based on the evidence presented in each article. It could very well be the case that the authors of the included studies have additional evidence of validity that we did not recognize as falling within these types of validities or that did not make it into their published articles.

### Conclusions

This is the first literature review that specifically identifies objective performance-based measurement tools of eHealth literacy in contrast to subjective self-report measurement tools. We identified 29 unique measurement tools of eHealth literacy with performance-based components used in various populations and covering various health-related topics. To better establish the utility and validity of performance-based measurement tools of eHealth literacy, scholars looking to incorporate performance-based measurement of eHealth literacy into their research should look to use and build from some of these existing measurement techniques rather than producing their own. In contexts where research participants can be provided a computer with internet access, measures from the *Health-related questions using the internet*, *Simulated internet tasks*, or *Website evaluation tasks* categories of this scoping review may offer ecologically valid options for assessing eHealth literacy. In contexts where providing such equipment may not be feasible, measures from the *Knowledge of the web-based health information–seeking process* category may offer a simpler performance-based eHealth literacy assessment tool that still reduces strict reliance on participants’ ability to gauge their own skill level.

In addition, to the best of our knowledge, this is the first literature review that quantifies the approximate prevalence of performance-based measurement versus subjective measurement of eHealth literacy in the literature broadly. In peer-reviewed scholarship, eHealth literacy assessment is predominantly conducted using subjective self-report measurement techniques. Performance-based measures of eHealth literacy are likely to provide a far better picture of how proficiently people find, evaluate, and use web-based health information. As such, researchers should consider using more objective measures of eHealth literacy such as those identified in this scoping review.

## Abbreviations

eHEALS: eHealth Literacy Scale
eHLA: eHealth literacy assessment toolkit
ERIC: Education Resources Information Center
LISA: Library and Information Science Abstracts
LISTA: Library, Information Science & Technology Abstracts
PRISMA-ScR: Preferred Reporting Items for Systematic Reviews and Meta-Analyses extension for Scoping Reviews
RRSA-h: Research Readiness Self-Assessment-health
